# Quality of Life in Patients with Venous Leg Ulcers Treated by Means of Local Hyperbaric Oxygen Therapy or Local Ozone Therapy—A Single Center Study

**DOI:** 10.3390/medicina59122071

**Published:** 2023-11-24

**Authors:** Jarosław Pasek, Sebastian Szajkowski, Grzegorz Cieślar

**Affiliations:** 1 Collegium Medicum im dr Władysława Biegańskiego, Jan Długosz University in Częstochowa, 13/15 Armii Krajowej St., 42-200 Częstochowa, Poland; 2 Faculty of Medical and Social Sciences, Warsaw Medical Academy of Applied Sciences, 8 Rydygiera St., 01-793 Warszawa, Poland; sebastianszajkowski@wp.pl; 3 Department of Internal Medicine, Angiology and Physical Medicine, Faculty of Medical Sciences in Zabrze, Medical University of Silesia in Katowice, 15 Stefana Batorego St., 41-902 Bytom, Poland; cieslar1@o2.pl

**Keywords:** quality of life, ozone therapy, local hyperbaric oxygen therapy, venous leg ulcers, treatment, hard-to-heal wounds

## Abstract

*Background and Objectives:* Venous leg ulcers pose a significant medical problem worldwide. The complexity of the problem determines the need for further interdisciplinary activities that will improve the quality of life for treated patients. This study compared the quality of life of patients with venous leg ulcers who received local hyperbaric oxygen therapy or local ozone therapy procedures as part of comprehensive treatment. *Materials and Methods:* The study included 129 patients (62 men and 57 women) with venous leg ulcers. Group I underwent local hyperbaric oxygen therapy (HBOT), and Group II underwent local ozone therapy (OZONE). In both groups, the patients’ quality of life was assessed before the start of the treatment cycle, as well as 10 weeks and 6 months after the completion of the treatment, by means of the EQ-5D-5L questionnaire and the Polish shortened version of the SF-36 scale. *Results:* After completing the respective therapeutic cycle, both groups showed statistically significant (*p* < 0.001) improvement in quality of life, according to the EQ-5D-5L questionnaire and the SF-36 scale. Differences were noted between the 1st examination (before treatment) and the 2nd examination (10 weeks after treatment), as well as the 3rd examination (6 months after treatment). In the EQ-5D-5L assessment of anxiety and depression, self-care, and activities of daily living 6 months after the end of treatment, better results were found in the group of patients treated with local hyperbaric oxygen therapy (*p* < 0.001). In this group, 6 months after the end of the treatment, a statistically significantly higher result on the EQ-VAS scale was also obtained (73.09 ± 19.8 points vs. 68.03 ± 17.37 points, *p* = 0.043). However, in the SF-36 assessment performed 6 months after the end of treatment, better results—a statistically significantly lower value of the quality of life index—were recorded in the group of patients treated with local ozone therapy (103.13 ± 15.76 points vs. 109.89 ± 15.42 points, *p* < 0.015). *Conclusions:* Hyperbaric oxygen therapy and local ozone therapy procedures have a beneficial effect on improving the quality of life of patients with venous leg ulcers.

## 1. Introduction

The assessment of quality of life (QoL), which is of interest for many fields of medicine, became popular only in the second half of the 20th century. Initially, it focused primarily on disabilities, which concerned chronic diseases. Over time, additional aspects began to be taken into account during the assessment, such as the patient’s emotional state, self-care, social activity, and cognitive abilities. Currently, quality of life is a complex and interdisciplinary issue. This concept is most fully defined by the commonly cited definition of the World Health Organization (WHO) as “*personal, individual perception of one’s own life position in the cultural context, the value system in which a person lives and in relation to the goals set, expectations, patterns and fears, the degree depending on the environment, social relations and environmental features*” [1,2].

Health-Related Quality of Life (HRQoL) is a much narrower concept than the general quality of life. It concerns the patient’s assessment of his or her current level of functioning and satisfaction in relation to his or her individual situation, depending on his or her current health condition. Health-related quality of life can be defined as “*the functional effect of an illness and its consequent therapy upon a patient, as perceived by the patient*” [3,4].

Despite significant progress in the intensive development of medicine and increasing expenditures made in regard to preventive measures and treatment, chronic wounds still constitute a significant problem for health care systems. It is estimated that approximately 1.5–2 million patients in Europe struggle with problems with difficult-to-heal wounds [5]. In the United States, this problem affects approximately 2.4–4.5 million people [6]. In Poland, leg ulcers occur in approximately 17–20% of the population. In over 50% of cases, leg ulcers persist for more than 9 months, and in over 2/3 of them, recurrences occur [7]. Ulcers are primarily the cause of chronic pain and limited mobility, which significantly reduces the sense of the quality of life of treated patients. The burdensome, painful nature of the disease, its chronicity, and frequent relapses force patients to actively seek help [8,9].

Leg ulcers of the lower limbs most often have a vascular etiology (more than 80% of all chronic leg ulcers), which is associated with venous insufficiency and atherosclerosis of peripheral arteries. They are more often diagnosed in women, and their incidence increases with age. The increase in the incidence of leg ulcers is also related to the demographic situation and aging of societies, as well as the increased incidence of lifestyle-related diseases such as obesity, diabetes, hypertension, and hypercholesterolemia, which are conducive to the development of chronic wounds and make their treatment more complicated [7,9,10].

The prevalence of the disease and the high costs of its treatment, often resulting from the inability to provide the care required for ulcers and an inappropriate lifestyle, make clinicians very interested in the search for new, more effective treatment methods. However, proper care for patients with leg ulcers does not only require knowledge of clinical data, but also, for example, mobility options, material conditions, family ties, the ability to change dressings, assessment of observed changes, and, above all, calls for appropriate selection of treatment methods that ensure effective improvement of the quality of life of these patients [11,12].

The aim of the study was to compare the quality of life of patients with venous ulcers of the lower limbs who received local hyperbaric oxygen therapy or local ozone therapy, using the EQ-5D-5L questionnaire and the shortened Polish version of the SF-36 scale.

## 2. Material and Methods

The study included 129 patients (62 men and 57 women) aged 55 to 78 years (average age: 67 years), diagnosed with venous leg ulcers, who were qualified on the basis of the following inclusion criteria: venous ulcers in the lower limbs in category C6 according to the CEAP classification, an ulcer surface area of over 5 cm^2^, partial damage to skin thickness (II° according to the NPIAP), ulcer persistence exceeding 6 weeks, an age range of >55 and <80 years, as well as informed and voluntary consent to participate in the study.

The exclusion criteria were as follows: ulcer etiology other than a venous one, ulcer surface area of less than 5 cm^2^, age in the range of <55 and >80 years, ulcer duration of less than 6 weeks, comorbidities (diabetes mellitus and hypertension), cognitive disorders, absence of voluntary and conscious patient’s consent to participate in the study.

The evaluation included patients hospitalized at the Department of Internal Diseases, Angiology, and Physical Medicine in Bytom, Poland, in the years 2019–2021, who were randomly divided into two groups differing in terms of the physical treatment method used. In group I, local hyperbaric oxygen therapy was used, and in group II, ozone therapy was used. In group I, consisting of patients treated with hyperbaric oxygen therapy, there were 36 men and 32 women (mean age 65.0 ± 10.9 years), whereas in group II, consisting of patients treated with local ozone therapy, there were 36 men and 25 women (mean age 66.0 ± 13.2 years). The average duration of leg ulceration in group I, which amounted to 3.36 ± 1.12 years, did not differ statistically significantly from the duration of leg ulceration in group II, which was 3.37 ± 1.25 years.

Local hyperbaric oxygen therapy procedures applied in group I were performed with the use of the LASEROBARIA-S device (FASSER S.A., Tarnowskie Góry, Poland) [13]. The treated limb was placed in the treatment chamber, which was sealed with an elastic collar at thigh level. The concentration of oxygen introduced into the chamber was about 95%; it was applied at a pressure of 1.5 ATA and a flow rate of about 5 L/min. The treatment cycle included 30 treatment procedures performed once a day, lasting 30 min each, applied in 2 series of 15 treatments (excluding Saturdays and Sundays). The interval between the two series of hyperbaric oxygen therapy was 4 weeks. The total length of the therapeutic cycle was 10 weeks.

Local ozone therapy procedures applied in group II were performed with the use of an Ato-3 device (Metrum Cryoflex, Blizne Łaszczyńskiego, Poland) [14]. Ozone was applied to the surface of the ulcer in the form of an oxygen-ozone mixture (5% ozone and 95% oxygen) with a concentration of 40 µg/mL, using the so-called “Ozone bag”. The duration of a single procedure was 30 min. Treatment procedures were also performed daily for 30 days in two series of 15 treatments (except for Saturdays and Sundays). The interval between two series of ozone therapy treatments was 4 weeks. The total length of the entire therapeutic cycle was 10 weeks.

During the cycles of combined physical treatment procedures performed in both groups of patients, a similar conventional pharmacological treatment was applied: micronized purified flavonoid fraction, pentoxifylline, and acetylsalicylic acid in standard doses. In addition, Allevyn Adhesive Ag dressing (Smith & Nephew Inc., Watford, Hertfordshire, UK) was topically applied to wounds. It ensures adequate moisture content and sterility of the wound, and it also exerts an antibacterial effect. After physical procedures, compression therapy was applied (compression class 3) for 17 h a day.

Before starting the cycle of physical treatment, after 10 weeks of therapy, and also 6 months after the end of treatment, a clinical assessment and questionnaire assessments of quality of life were performed in both groups.

The Polish version of the EQ-5D-5L questionnaire was used to assess the quality of life of the examined patients. The EQ-5D-5L survey is a standardized document, and it did not require any validation. The survey assessed 5 dimensions of quality of life: moving around (1), self-care (2), performing ordinary everyday activities such as work, studying, and household chores (3), feeling pain/discomfort (4), and feeling anxious/depressed (5). Dimensions were assessed using a 5-point Likert scale. The respondents were asked to mark with an “x” one of the following levels that best reflected their current health condition: 1—no problems; 2—minor problems/slight severity; 3—moderate problems/moderate severity; 4—serious problems/severe severity; and 5—inability to perform activities/very severe intensity.

Additionally, an assessment was performed using the EuroQoL Visual Analogue Scale (EQ-VAS), with which the patient assessed his or her current health condition on a scale ranging from 0 (worst imaginable state) to 100 (best imaginable state) [15].

The second research tool that was used was a shortened Polish version of the SF-36 quality of life questionnaire assessing 8 dimensions of health, i.e., physical functioning, social functioning, role limitation in relation to physical problems, role limitation in relation to emotional problems, mental health, vitality, pain, and general health assessment. The categories are grouped into two scales: the physical one (PCS—Physical Component Summary—1) and the mental one (MCS—Mental Component Summary—2). The quality of life index (QoL index—3) is the sum of points scored when assessing all 8 dimensions of quality of life, and it allows for an overall assessment of health status. According to the Polish version, the more points scored, the more negative the subject’s self-esteem concerning his/her quality of life [16].

The assessment was performed by people with medical education (a doctor and a physiotherapist) with competences in assessing health conditions and the effectiveness of the wound treatment process.

The study was conducted in accordance with the Declaration of Helsinki (1964), and its protocol was accepted by the local bioethical committee at the Medical University of Silesia in Katowice, Poland (approval reference number: PCN/0022/KB1/102/III/16/19/21 dated 19 January 2021). All enrolled patients gave written informed consent for participation in the study and agreed to complete the questionnaire.

### Statistical Analysis

The Statistica software package version 13 (StatSoft Polska Sp. z o.o., Kraków, Poland) was used to analyze the collected results. Those results were presented using mean values, standard deviations, and 95% confidence intervals. The distribution of the variables studied was examined using the Shapiro–Wilk test. Due to the compliance with normal distribution, the Student’s *t*-test and one-way analysis of variance (ANOVA) were used to test the statistical significance of differences in the examined parameters. The level of statistical significance was assumed to be *p* < 0.05.

## 3. Results

The assessment of the quality of life of the study subjects, performed before the beginning of treatment using the EQ-5D-5L questionnaire, shows high score values for all assessed dimensions of quality of life, which confirms that quality of life was at a low ebb in these patients.

The analysis of results obtained in assessing the quality of life of the examined patients, performed according to the EQ-5D-5L questionnaire, shows a statistically significant improvement in results of quality of life assessment (*p* < 0.001) for all five dimensions of quality of life considered, both in the group of patients who received local hyperbaric oxygen therapy and in the group of patients who received local ozone therapy. In both compared groups of patients, a statistically significant improvement (lower score) was noted between study 1 (before the start of treatment) and study 2 (after 10 weeks of treatment), as well as study 3 (6 months after the end of treatment) (Table 1).

**Figure 1:**
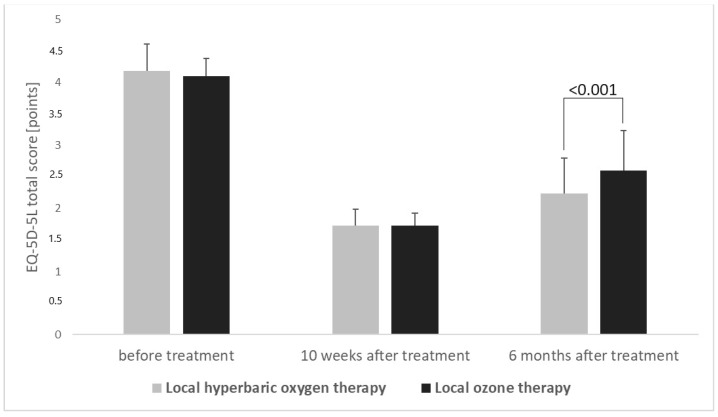
Total scores obtained by means of evaluation using the EQ-5D-5L questionnaire (mean and SD values) concerning the treatment of patients by means of local hyperbaric oxygen therapy and local ozone therapy, as assessed during the 6-month follow-up.

Figure 1 presents the evaluation of total scores obtained by means of the EQ-5D-5L questionnaire in groups of patients treated by means of local hyperbaric oxygen therapy and local ozone therapy in the period from the beginning of therapy to 6 months after completion of the therapy.

The detailed analysis of the individual dimensions of quality of life, assessed according to the study utilizing the EQ-5D-5L questionnaire conducted after 10 weeks of treatment, shows a statistically significantly lower score regarding the feeling of anxiety and depression in the group of patients treated by means of local hyperbaric oxygen therapy, as compared to the group of patients treated applying local ozone therapy (1.50 ± 0.50 points vs. 1.67 ± 0.47 points, *p* < 0.048), which was associated with a larger degree of mood improvement in this group of patients (Table 1).

In turn, the analysis of individual dimensions of quality of life, assessed according to the EQ-5D-5L questionnaire carried out 6 months after the end of treatment in the group of patients treated by means of local hyperbaric oxygen therapy, when compared to the group of patients treated by means of local ozone therapy, shows a statistically significantly lower score regarding the overall assessment of quality of life (2.24 points vs. 2.60 points, *p* < 0.001), as well as statistically significantly lower scores regarding, respectively, self-service efficiency (2.06 ± 0.83 points vs. 2.56 ± 0.83 points, *p* < 0.001), performing ordinary everyday activities (2.07 ± 0.75 points vs 2.57 ± 0.88 points, *p* < 0.001), feeling pain and discomfort (2.00 ± 0.64 points vs. 2.31±0.82 points, *p* = 0.018), and feeling anxiety and depression (1.92 ± 0.55 points vs 2.27 ± 0.93 points, *p* = 0.009), which indicates greater therapeutic effectiveness of hyperbaric oxygen therapy in terms of improving the assessment of the above dimensions of quality of life in treated patients (Table 1).

It should be emphasized that in both groups of patients that were compared, the scores for individual dimensions of quality of life 6 months after the end of treatment were higher than after 10 weeks of treatment, which indicates that the therapeutic effect obtained was transient, not durable.

The analysis of the test results regarding pain symptoms assessed using the EQ-VAS scale carried out after 10 weeks of treatment shows a statistically significant reduction in the intensity of pain sensation in both groups of patients (*p* < 0.001), while the results obtained in both compared groups did not differ statistically significantly (*p* = 0.122). However, in the analysis conducted 6 months after the end of treatment, a statistically significantly higher score was recorded in the group of patients treated by means of local hyperbaric oxygen therapy as compared to the group of patients treated by means of ozone therapy (73.09 ± 19.80 points vs. 68.03 ± 17.37 points, *p* = 0.043).

The assessment of the quality of life of the study subjects, carried out before the start of treatment and performed using the SF-36 questionnaire, shows high score values both in the physical and mental scales and in the case of the quality of life index, which confirms the quality of life was low in these patients.

The analysis of the results of quality of life assessment of the study subjects carried out according to the SF-36 questionnaire also shows a statistically significant improvement in the results of the quality of life assessment (lower score) (*p* < 0.001) in the assessed physical and mental spheres and in the quality of life index, both in the group of patients who received local hyperbaric oxygen therapy as well as in the group of patients who received local ozone therapy. In both groups of patients that were compared, a statistically significant improvement (lower score) was noted between study 1 (before the start of treatment) and study 2 (after 10 weeks of treatment), as well as study 3 (6 months after the end of treatment) (Table 2).

A detailed analysis of the assessment of quality of life by means of the SF-36 scale carried out after 10 weeks of treatment did not show statistically significant differences between the groups of patients treated with the use of both physical methods compared, in regard to the scores recorded on the physical and mental scales, but shows a statistically significantly lower value of the quality of life index scores in case of patients treated by means of local ozone therapy, as compared to patients treated by means of local hyperbaric oxygen therapy (79.37 ± 8.99 points vs. 85.39 ± 8.96 points); (*p* < 0.001), which indicates a higher therapeutic effectiveness of local ozone therapy in this respect (Table 2).

Similarly, the analysis of the assessment of quality of life using the SF-36 scale carried out 6 months after the completion of treatment did not show statistically significant differences between the groups of patients treated with the use of both physical methods compared, as concerns the scores on the physical and mental scales, but showed a statistically significantly lower value of the quality of life index scores in patients treated by means of local ozone therapy, compared to patients treated by means of local hyperbaric oxygen therapy (103.13 ± 15.76 points vs. 109.89 ± 15.42 points, *p* = 0.015), which indicates that the therapeutic effectiveness of local ozone therapy remains higher in this respect as well as in long-term observation (Table 2).

It should be emphasized that in both groups of patients that were compared, the values of the quality of life index assessed 6 months after the end of treatment were higher than after 10 weeks of treatment, which indicates that the therapeutic effect obtained was not so durable.

## 4. Discussion

The quality of life of patients has been the subject of many assessments and analyses over several decades. The undertaking of therapeutic activities is meant to improve the quality of life of a sick person who has to struggle with the distressing symptoms of the disease, i.e., pain, functional limitations, and self-care. The quality of life is determined by seeing it through the prism of various areas of life, although one of its most important determinants is the generally understood well-being, which results from assessing one’s health, both physical and mental [1,17,18].

When assessing the effects of the treatment of venous leg ulcers (VLU), attention should be paid not only to the elimination of the symptoms of the disease, but also to the biopsychosocial sphere of the patient’s functioning, including the ability to perform self-care and fulfill specific social roles, as well as the subjective assessment of the quality of life. When assessing the quality of life in the case of VLU, the authors have used a number of both general and disease-specific research tools. An aspect that has not yet been fully resolved is the determination of the features that are characteristic of VLU, which are the most decisive ones for the reduction of health-related quality of life (HRQoL). Most authors consider pain, the size of the wound surface area, discharge from the wound, and unpleasant odor to be the key factors reducing HRQoL in these patients. [4,19,20,21].

To assess whether the medical care provided to patients with VLU contributes to improving their overall quality of life, the first step should be, as mentioned above, to identify factors related to the quality of life in relation to health [22]. So far, as indicated by the research conducted by Hsiao et al., in some countries (e.g., Taiwan), there have been no studies carried out that pertain to the quality of life of patients with VLU [23].

It should be emphasized that patients with difficult-to-heal wounds face such problems as reduced physical fitness, the need to change their current lifestyle, changes in their own preferences, as well as changes in the family and social environment. In addition, the inferior quality of life is related to the presence of wound exudate, unpleasant odor, pain, insomnia, depression, and anxiety, which result in difficult functioning at home, work, and community, the deterioration of interpersonal relationships, a lack of acceptance, confusion, lowered self-esteem, and the inability to perform current roles in social conditions. In many cases, it also forces people with VLUs to resign from paid employment, resulting in the doom of social isolation [24,25]. The deterioration in the quality of life occurring in patients with venous leg ulcers caused by the above factors was confirmed by the results of our research, which revealed unfavorable scores in both quality assessment questionnaires used to examine both groups of patients regarding both the physical and mental spheres, encompassing all assessed dimensions of quality of life.

The inferior quality of life in patients with venous leg ulcers was also confirmed by the authors of numerous publications available.

Miertová et al. assessed selected aspects of quality of life in 61 patients with venous leg ulcers treated in outpatient settings using the modified Freiburg Life Quality Assessment wound module (FLQA-w) questionnaire. The most disappointing result was obtained in the category of everyday life (3.61 ± 0.93 points), and a significant impact on the outcome of the assessment of the quality of life, among others, was related to the duration of ulcer treatment [26].

Iglesias et al. assessed HRQoL in 387 patients with venous leg ulcers from nine regions of the UK, using the SF-12, EQ-5D-5L, and Hyland questionnaires. According to the authors, the SF-12 and EQ-5D-5L questionnaires revealed good assessment properties and were responsive to changes in the HRQoL assessment after ulcer healing [27].

Dias et al. compared the quality of life in 204 patients with chronic venous disease using the SF-36 questionnaire and showed that in the group of patients with venous ulcers, the scores for all aspects of quality of life were significantly lower [24].

González et al. assessed HRQoL in 34 patients with venous leg ulcers lasting for 3–5 months, taking the stage of ulceration into account. They showed that venous ulcers have a negative impact, especially on the emotional state, and the presence of tissue with poor regenerative capacity and poor control of exudate and infection determine the deterioration of HRQoL results [11].

Hareendran et al. assessed HRQoL in a group of 38 patients with venous leg ulcers. They found that significantly worse HRQoL assessment results (*p* < 0.05) occurred in elderly patients with pain and non-healing wounds. The factors contributing to the deterioration of HRQoL assessment results included pain (80.5%); pruritus (69.4%); changed skin appearance (66.7%); sleeplessness (66.6%); functional limitations (58.3%); and disappointment with the effects of treatment (50%) [28].

Folguera-Alvarez et al., after conducting a cross-sectional study in 22 primary health care centers on a sample of 93 patients with VLU, showed that the severity of the ulcer, pain, and symptoms of infection significantly reduced the perceived quality of life of the studied patients [29].

Van Korlaar et al., in turn, after conducting a query involving 25 articles from available databases that aimed to assess the quality of life of patients with chronic venous diseases, found that the quality of life of patients with chronic venous disease is disturbed, especially in the physical domain, mainly in relation to pain, physical functioning, and mobility. The results also showed the occurrence of negative emotional reactions and social isolation of the respondents [30].

Szewczyk et al., based on research conducted in 2005, found that over 80% of patients with chronic leg ulcers give up developing their own interests and reduce the frequency of participating in social gatherings and making new friends due to distressing symptoms. Moreover, pain associated with ulcers caused insomnia, fatigue, exhaustion, and a lack of energy, which resulted in the frequent occurrence of depression [31].

In another study, Jawień et al. assessed the biopsychosocial functioning and occurrence of limitations in people with varicose veins of the lower leg. The authors found a frequent occurrence of depression in the group of patients studied, as well as a higher risk of falls and malnutrition. Moreover, patients faced an increased risk of impaired functioning in the social, emotional, and physical spheres [32].

Then, Ścisło et al. assessed the quality of life of 61 patients with venous ulcers of the lower limbs. The surveyed patients rated their satisfaction with the quality of life higher than their objective health status. They gave the highest rating to the quality of life in the social area and the lowest to the physical area [33].

In the study reported here, the EQ-5D-5L questionnaire was used to assess the quality of life, which is characterized by high sensitivity and precision and, at the same time, is understandable to patients. Most authors believe that increasing the number of levels from 3L to 5L in each dimension increased the “sensitivity” of the EQ-5D-5L questionnaire. Another advantage of the EQ-5D-5L questionnaire used in this study is its property of universality and applicability to any medical condition. On the other hand, the EQ-5D-5L questionnaire also has its limitations. First of all, it does not cover spiritual aspects, although this area is now considered very important in the provision of holistic patient care [15,34].

The aim of the study was to assess the quality of life of patients who received treatment in the form of two physical methods, namely local hyperbaric oxygen therapy or local ozone therapy. The quality of life of the study subjects was assessed before the start of treatment, 10 weeks after the start of treatment, as well as 6 months after the completion of treatment. As shown by the research results, in each of the evaluations, positive results were obtained, showing the beneficial impact of the physical methods used upon the quality of life of the examined patients regarding both the physical and mental spheres, as well as all assessed dimensions of quality of life. Since in both groups of patients compared, the scores for all dimensions of quality of life assessed using the EQ-5D-5L questionnaire and the quality of life index assessed using the SF-36 questionnaire deteriorated 6 months after the completion of treatment, being worse than immediately after the end of the 10-week treatment cycle, it should be assumed that the therapeutic effect obtained with the use of both compared therapeutic methods, in relation to the assessment of patients’ quality of life, was not fully sustainable.

In the analysis of long-term therapeutic effects achieved using both physical methods discussed, the group of patients treated with local hyperbaric oxygen therapy was compared to the group of patients treated with local ozone therapy. In the case of a detailed analysis of individual dimensions of quality of life according to the EQ-5D-5L questionnaire, a statistically significantly lower score was shown regarding efficiency in self-care, performing ordinary everyday activities, feeling pain and discomfort, as well as feeling anxiety and depression. In the case of the analysis using the SF-36 questionnaire, a statistically significantly higher score of the quality of life index was noted, which indicates greater therapeutic effectiveness of local hyperbaric oxygen therapy in terms of improving the life quality dimensions analyzed in the EQ-5D-5L questionnaire. For local ozone therapy, the improvement concerned the quality of life index of patients treated for venous leg ulcers, as analyzed by means of the SF-36 questionnaire.

The available literature contains only a few publications assessing the impact of the therapy on improving the quality of life of patients with venous leg ulcers.

In the study by Tiwary et al., quality of life was assessed in 50 patients with venous leg ulcers by means of the SF-36, Charing Cross Venous Ulcer Questionnaire (CXVUQ), and the Chronic Venous Disease Clinical Severity Scale (VCSS). After endovascular treatment and compression therapy, the ulcers healed in most but seven (12.5%) patients. A recurrence of ulcers was observed in 12 limbs (21.43%). However, the quality of life assessed after the completion of treatment showed a statistically significant improvement compared to the baseline values from before the start of therapy (*p* < 0.001) [35].

In a retrospective, single-center cohort study performed by Lalieu et al., 15 patients from an outpatient clinic with VLUs were treated with HBOT and standard wound care. Patients received an average of 43 ± 20 sessions of HBOT. The improvement in patient-related outcome measures (PROMs) was assessed by the EQ-5D-3L questionnaire and included quality of life (QoL) and pain scores. Most of those patients reported improvement in all health aspects covered by the questionnaire—the pain score decreased from 5.7 (±2.5) to 2.1 (±2.2) (*p* < 0.0001), and the health score increased from 57.2 ± 15.6) to 69.9 ± 18.9) (*p* = 0.02) [36].

Morteza et al. evaluated the effect of ozone therapy on the health-related quality of life of 86 patients with chronic wounds. To measure the quality of life, the Cardiff wound impact questionnaire and the SF-36 questionnaire were used. As an effect of medical O_3_ therapy, a significant improvement in the healing of chronic wounds as well as a significant improvement in the health-related quality of life of patients were observed. According to the authors, this method of treatment could be a valuable therapeutic option in the treatment of chronic wounds [37].

The confirmation of beneficial effects of therapy on the assessed quality of life was also obtained in the work of Solidade-Simoes et al., in which the quality of life was assessed in 171 patients with venous leg ulcers treated in primary health care units in two cities in Brazil and Portugal. The quality of life scores measured using a shortened version of the SF-36 questionnaire were significantly better in Portugal concerning physical aspects, pain, and social functioning in different domains, as well as the physical health dimension and total QoL score [38].

The results of the studies presented above confirm the beneficial effect of treatment with physical methods, such as local hyperbaric oxygen therapy and local ozone therapy, on the assessed quality of life of patients with venous leg ulcers, as determined in our study.

The basis for proper care of patients with leg ulcers (regardless of their etiology) is to minimize the loss of functionality, maintain the ability to perform basic daily activities independently, and prevent and protect against deterioration of quality of life as a result of the disease. An important aspect of that is also the individual planning of the educational process. Likewise, it is also vital to state that the psychosocial costs associated with venous leg ulcers are relatively high. The association of venous leg ulcers with anxiety, pain, depression, social isolation, and sleep disorders invariably manifests itself in a reduced quality of life. Hence, conducting research in this area is becoming increasingly important. The data obtained from quality of life studies can help take clinical decisions and determine health strategies for the treatment of patients with difficult-to-heal wounds, and the results of these studies will allow medical care providers to focus not only on the factors influencing ulcer healing, but also on the factors affecting patients’ HRQoL, as a holistic assessment of the needs of patients with VLU is currently recommended in order to conduct optimal and cost-effective treatment [39].

### Limitations of the Study

With the above in mind, it is also important to acknowledge the limitations of the study, such as the non-representative and relatively small sample size, the lack of a control group receiving standard care only, and the recruitment of participants only from one health center. The study also did not include an analysis of previous standards of care.

## 5. Conclusions

Therapy using local hyperbaric oxygen therapy as well as local ozone therapy appears to have a positive effect on improving the quality of life of patients suffering from venous leg ulcers treated with the use of those therapies in the 6-month observation period. This improvement concerns, in particular, the mental sphere, self-service, and performing everyday activities, as well as the feelings of pain and mobility. The research that was conducted shows that none of the physical methods compared shows a clear advantage in terms of therapeutic effectiveness in this area, and the observed differences result from the very method that was used to assess the quality of life. Developing and implementing wound care strategies that also focus on improving health-related quality of life is a challenge for modern healthcare systems around the world. Therefore, it seems reasonable to continue research concerning the analysis of the quality of life parameters assessed using a larger group of study subjects.

## Figures and Tables

**Table 1:** The assessment of quality of life according to the EQ-5D-5L questionnaire [points scored] in patients treated with hyperbaric oxygen therapy and local ozone therapy, along with statistical evaluation.

Quality of Life Dimension and Assessment Period	Local Hyperbaric Oxygen Therapy (*n* = 68)				Local Ozone Therapy (*n* = 61)				*p*
	Mean	Confidence −95%	Confidence 95%	SD	Mean	Confidence −95%	Confidence 95%	SD	
EQ-5D-5L (1) before treatment	4.57	4.45	4.70	0.53	4.46	4.33	4.59	0.50	0.210
EQ-5D-5L (2) before treatment	3.82	3.58	4.07	1.02	3.82	3.62	4.02	0.76	0.981
EQ-5D-5L (3) before treatment	3.96	3.78	4.14	0.74	3.79	3.64	3.94	0.58	0.155
EQ-5D-5L (4) before treatment	4.44	4.31	4.57	0.53	4.31	4.17	4.45	0.53	0.169
EQ-5D-5L (5) before treatment	4.15	4.00	4.29	0.61	4.16	4.02	4.31	0.55	0.869
EQ-5D-5L (1) 10 weeks after treatment	2.24	2.10	2.37	0.55	2.26	2.14	2.39	0.48	0.768
EQ-5D-5L (2) 10 weeks after treatment	1.69	1.57	1.81	0.50	1.52	1.40	1.65	0.50	0.061
EQ-5D-5L (3) 10 weeks after treatment	1.62	1.50	1.74	0.49	1.57	1.45	1.70	0.50	0.615
EQ-5D-5L (4) 10 weeks after treatment	1.65	1.53	1.76	0.48	1.66	1.53	1.78	0.48	0.919
EQ-5D-5L (5) 10 weeks after treatment	1.50	1.38	1.62	0.50	1.67	1.55	1.79	0.47	**0.048**
EQ-5D-5L (1) 6 months after treatment	3.18	2.93	3.43	1.04	3.33	3.13	3.52	0.77	0.352
EQ-5D-5L (2) 6 months after treatment	2.06	1.86	2.26	0.83	2.56	2.35	2.77	0.83	**0.001**
EQ-5D-5L (3) 6 months after treatment	2.07	1.89	2.26	0.76	2.57	2.35	2.80	0.88	**0.001**
EQ-5D-5L (4) 6 months after treatment	2.00	1.84	2.16	0.65	2.31	2.10	2.52	0.83	**0.018**
EQ-5D-5L (5) 6 months after treatment	1.93	1.79	2.06	0.55	2.28	2.04	2.52	0.93	**0.009**
EQ-VAS before treatment	40.27	37.07	43.57	21.33	38.10	37.2	39	22.71	0.569
EQ-VAS 10 weeks after treatment	66.19	62.69	69.69	24.79	63.62	61.82	65.42	21.08	0.122
EQ-VAS 6 months after treatment	73.09	71.79	74.49	19.80	68.03	64.93	71.13	17.37	**0.043**

(1) moving around; (2) self-care; (3) performing normal activities of daily living; (4) pain/discomfort; (5) anxious/depressed. *p* value in bold indicates statistical significance.

**Table 2:** The assessment of the quality of life according to the SF-36 [points scored] questionnaire in patients treated by means of hyperbaric oxygen therapy and local ozone therapy with statistical evaluation.

Quality of Life Scale and Assessment Period	Local Hyperbaric Oxygen Therapy (*n* = 68)				Local Ozone Therapy (*n* = 61)				*p*
	Mean	Confidence −95%	Confidence 95%	SD	Mean	Confidence −95%	Confidence 95%	SD	
SF-36 (1) before treatment	81.21	78.56	83.86	10.95	84.25	81.92	86.57	9.08	0.091
SF-36 (2) before treatment	59.32	58.31	60.34	4.19	59.54	58.48	60.61	4.16	0.768
SF-36 (3) before treatment	144.21	140.83	147.58	13.93	146.20	142.99	149.40	12.51	0.397
SF-36 (1) 10 weeks after treatment	50.72	48.88	52.56	7.62	50.07	48.30	51.83	6.90	0.611
SF-36 (2) 10 weeks after treatment	40.24	38.46	42.01	7.33	39.75	38.10	41.41	6.46	0.694
SF-36 (3) 10 weeks after treatment	85.40	83.23	87.57	8.96	79.38	77.07	81.68	8.99	**0.001**
SF-36 (1) 6 months after treatment	58.99	56.78	61.19	9.11	61.11	59.11	63.12	7.82	0.159
SF-36 (2) 6 months after treatment	50.04	48.70	51.39	5.56	51.89	49.23	54.54	10.37	0.205
SF-36 (3) 6 months after treatment	109.90	106.16	113.63	15.42	103.13	99.09	107.17	15.76	**0.015**

(1) physical scale; (2) mental scale; (3) quality of life index. *p* value in bold indicates statistical significance.

## Data Availability

The datasets used and/or analyzed during the current study are available from the corresponding author on reasonable request.
